# *Ilex paraguariensis* modulates fat metabolism in *Caenorhabditis elegans* through purinergic system (ADOR-1) and nuclear hormone receptor (NHR-49) pathways

**DOI:** 10.1371/journal.pone.0204023

**Published:** 2018-09-25

**Authors:** Marina Lopes Machado, Leticia Priscilla Arantes, Priscila Gubert, Daniele Coradini Zamberlan, Thayanara Cruz da Silva, Tássia Limana da Silveira, Aline Boligon, Félix Alexandre Antunes Soares

**Affiliations:** 1 Departamento de Bioquímica e Biologia Molecular, Programa de Pós-graduação em Ciências Biológicas: Bioquímica Toxicológica, Universidade Federal de Santa Maria, Santa Maria, Rio Grande do Sul, Brazil; 2 Centro de Ciências Biológicas e da Saúde, Campus Reitor Edgard Santos, Universidade Federal do Oeste da Bahia, Barreiras, Bahia, Brazil; 3 Departamento da Farmácia Industrial, Laboratório de Pesquisa Fitoquímica, Universidade Federal de Santa Maria, Santa Maria, Rio Grande do Sul, Brazil; East Carolina University, UNITED STATES

## Abstract

*Ilex paraguariensis* is a well-known plant that is widely consumed in South America, primarily as a drink called *mate*. *Mate* is described to have stimulant and medicinal properties. Considering the potential anti-lipid effects of *I*. *paraguariensis* infusion, we used an extract of this plant as a possible modulator of fat storage to control lipid metabolism in worms. Herein, the *I*. *paraguariensis*-dependent modulation of fat metabolism in *Caenorhabditis elegans* was investigated. *C*. *elegans* were treated with *I*. *paraguariensis* aqueous extract (1 mg/ml) from L1 larvae stage until adulthood, to simulate the primary form of consumption. Expression of adipocyte triglyceride lipase 1 (ATGL-1) and heat shock protein 16.2, lipid accumulation through C1-BODIPY-C12 (BODIPY) lipid staining, behavioral parameters, body length, total body energy expenditure and overall survival were analyzed. Total body energy expenditure was determined by the oxygen consumption rate in N2, nuclear hormone receptor knockout, *nhr-49(nr2041)*, and adenosine receptor knockout, *ador-1(ox489)* strains. *Ilex paraguariensis* extract increased ATGL-1 expression 20.06% and decreased intestinal BODIPY fat staining 63.36%, compared with the respective control group, without affecting bacterial growth and energetic balance, while *nhr-49(nr2041)* and *ador-1(ox489)* strains blocked the worm fat loss. In addition, *I*. *paraguariensis* increased the oxygen consumption in N2 worms, but not in mutant strains, increased N2 worm survival following juglone exposure, and did not alter *hsp-16*.*2* expression. We demonstrate for the first time that *I*. *paraguariensis* can decrease fat storage and increase body energy expenditure in worms. These effects depend on the purinergic system (ADOR-1) and NHR-49 pathways. *Ilex paraguariensis* upregulated the expression of ATGL-1 to modulate fat metabolism. Furthermore, our data corroborates with other studies that demonstrate that *C*. *elegans* is a useful tool for studies of fat metabolism and energy consumption.

## 1. Introduction

A number of plants are used as complementary or alternative medicines in regular diets worldwide [1]. *Ilex paraguariensis* St. Hil. Var. *paraguariensis* (Aquifoliaceae), the *yerba mate*, is widely used in southern Brazil, northern Argentina, Paraguay, and Uruguay [2] as a drink called *chimarrão*, *tererê*, *or mate*. Its consumption has been popular for centuries because/ of its stimulant and medicinal properties [3]. The effects of the consumption of *I*. *paraguariensis* include central nervous system stimulation [4], increased antioxidant defense [5], antioxidant properties *in vitro* [6], and thermogenic properties [7].

The prevalence of obesity is increasing worldwide, and has drawn the attention of public health institutions, as it is commonly associated with various metabolic disorders such as hypertension, dyslipidemia, type II diabetes, and insulin resistance [8]. In 2011–2012, 34.9% of adults aged 20 years and over were obese in the United States of America [9], indicating the urgent need for new treatments. Many methods are used to treat obesity, most of which are pharmaceuticals, which can cause collateral effects, like psychiatric disorders, heart attack, and stroke [10], and are often associated with rebound weight gain and potential drug abuse [11]. In this context, natural extracts are potential alternatives to pharmaceuticals and should be investigated for new anti-obesity treatments or for nutraceutical prevention for obesity, as they often cause less adverse effects and are easily added to the diet [12].

Previous studies have reported that the main compound found in aqueous extracts of *I*. *paraguariensis* are methylxanthines. The primary methylxanthine is caffeine [13, 14], which is a thermogenic agent that acts through the adenosine receptor [15] to increase metabolic rates [16–18], induce fat oxidation [19–21], stimulate respiratory centers [16], and increase resting energy expenditure [22].

Herein, we studied the modulation of fatty acid metabolism by *I*. *paraguariensis* extract *in vivo* using *Caenorhabditis elegans* as an animal model. This nematode has been described as a widely accepted and used model organism for studies of a variety of biological processes and diseases that can be defined on a molecular basis, e.g., obesity and fat metabolism, because many proteins involved in the synthesis, oxidation, and transport of lipids are highly conserved between *C*. *elegans* and mammals [23].

In *C*. *elegans*, adenosine receptor ortholog (ADOR-1), nuclear hormone receptor (NHR-49), and adipose triglyceride lipase (ATGL-1) can be studied to evaluate the purinergic system [24], the regulation of β-oxidation [25] rate-limiting genes, and fat mobilization from stored triglycerides (TAGs) [26], respectively. This study aimed to investigate whether these pathways are involved in *I*. *paraguariensis* modulation of fat metabolism in *C*. *elegans*.

## 2. Materials and methods

### 2.1. Chemical, apparatus and general procedures of analytical grade

Methanol, formic acid, gallic acid, chlorogenic acid and caffeic acid were purchased from Merck (Darmstadt, Germany). Quercetin, theobromine, caffeine, rutin, catechin, epigallocatechin and kaempferol were acquired from Sigma Chemical Co. (St. Louis, MO, USA). High performance liquid chromatography (HPLC-DAD) was performed with a Shimadzu Prominence Auto Sampler (SIL-20A) HPLC system (Shimadzu, Kyoto, Japan), equipped with Shimadzu LC-20AT reciprocating pumps connected to a DGU 20A5 degasser with a CBM 20A integrator, SPD-M20A diode array detector and LC solution 1.22 SP1 software.

### 2.2. Plant material and aqueous extract preparation

Minced leaves of *Ilex paraguariensis* from *Ervateira Seiva-Pura*^®^ used in this study were purchased from local market in Santa Maria, Rio Grande do Sul (Brazil). The extraction was carried out by pouring 100 mL of boiled distilled water on plant sample [27]. After extraction at room temperature (10 min), the aqueous extract was filtered using a sterilization filter with 0.22μm pore size.

### 2.3. Quantification of compounds by HPLC-DAD

Reverse phase chromatographic analyses were carried out under gradient conditions using C18 column (4.6 mm x 250 mm) packed with 5μm diameter particles. The mobile phase was water containing 1% formic acid (A) and methanol (B), and the composition gradient was: 15% of B until 10 min and changed to obtain 20%, 30%, 50%, 60%, 70%, 20% and 10% B at 20, 30, 40, 50, 60, 70 and 80 min, respectively, following the method described by Abbas et al. (2014) with slight modifications [28]. *Ilex paraguariensis* aqueous extract was analyzed at a concentration of 20 mg/Mr. The presence of ten compounds was investigated: Gallic acid, chlorogenic acid, caffeic acid, catechin, epigallocatechin, quercetin, rutin, kaempferol, caffeine and theobromine. Identification of these compounds was performed by comparing their retention time and UV absorption spectrum with those of the commercial standards. The flow rate was 0.7 ml/min, injection volume 40 μL and the wavelength were 257 nm for gallic acid, 270 nm for theobromine, 280 nm for catechin, epigallocatechin and caffeine, 327 nm for caffeic and chlorogenic acids, and 366 nm for quercetin, rutin and kaempferol (Table 1). All the samples and mobile phase were filtered through 0.45 μm membrane filter (Millipore) and then degassed by ultrasonic bath prior to use. Stock solutions of standards references were prepared in the HPLC mobile phase at a concentration range of 0.030–0.250 mg/ml for kaempferol, quercetin, catechin, epigallocatechin, rutin, caffeine and theobromine; and 0.045–0.300 mg/ml for gallic, caffeic and chlorogenic acids. The chromatography peaks were confirmed by comparing its retention time with those of reference standards and by DAD spectra (200 to 600 nm). Calibration curve for gallic acid: Y = 13057x + 1285.4 (r = 0.9998); catechin: Y = 12728x + 1197.5 (r = 0.9995); epigallocatechin: Y = 11893 + 1357.2 (r = 0.9995); chlorogenic acid: Y = 12659x + 1287.8 (r = 0.9993); caffeic acid: Y = 11962x + 1326.2 (r = 0.9997); caffeine: Y = 13276x + 1297.6 (r = 0.9999); theobromine: Y = 12473x + 1175.8 (r = 0.9996); rutin: Y = 13805 + 1195.7 (r = 0.9999); quercetin: Y = 13627x + 1362.1 (r = 0.9999) and kaempferol: Y = 12583x + 1274.8 (r = 0.9997). The limit of detection (LOD) and limit of quantification (LOQ) were calculated based on the standard deviation of the responses and the slope using three independent analytical curves. LOD and LOQ were calculated as 3.3 and 10 σ/S, respectively, where σ is the standard deviation of the response and S is the slope of the calibration curve [29].

**Table 1:** Composition of *Ilex paraguariensis* aqueous extracts.

Compounds	*Ilex paraguariensis*		LOD	LOQ
	mg/g	%	μg/mL	μg/mL
Gallic acid	1.27 ± 0.01^a^	0.12	0.015	0.049
Catechin	2.98 ± 0.03^b^	0.29	0.032	0.105
Chlorogenic acid	3.71 ± 0.01^b^	0.37	0.008	0.027
Caffeic acid	9.15 ± 0.02^c^	0.91	0.021	0.070
Caffeine	8.86 ± 0.01^d^	0.88	0.029	0.095
Theobromine	3.65 ± 0.01^b^	0.36	0.007	0.023
Epigallocatechin	6.01 ± 0.03^e^	0.60	0.016	0.052
Rutin	7.43 ± 0.02^f^	0.74	0.026	0.086
Quercetin	3.12 ± 0.01^b^	0.31	0.035	0.115
Kaempferol	5.95 ± 0.03^e^	0.59	0.019	0.063

Results are expressed as the mean ± standard deviation (SD) of three determinations.

Averages followed by different letters differ by Tukey test at p < 0.05.

LOD, limit of detection; LOQ, limit of quantification.

### 2.4. *C*. *elegans* strains

Wild-type *C*. *elegans* strain N2 wild-type (var. Bristol), STE68 *nhr-49(nr2041)*, VS20 (*hjIs67[atgl-1p*::*atgl-1*::*gfp + mec-7*::RFP]) and CL2070 *dvIs70 Is[hsp-16*.*2*::*gfp; rol-6(su1006)]* were provided by the *Caenorhabditis* Genetics Center (University of Minnesota, USA). EG6870 strain, *ador-1(ox489)*, was kindly supplied from Dr. Erik Jorgensen laboratory (University of Utah, USA). This strain has a deletion from 1kb upstream and the first three exons of the *ador*-1 gene, and was outcrossed six times. All strains were maintained at 20°C.

### 2.5. Growth conditions and *Ilex paraguariensis* treatment

Treatment plates were prepared diluting *Ilex paraguariensis* aqueous extract in distilled autoclaved water and spreading it with *Escherichia coli* OP50 as food source to the surface of dry nematode grow media (NGM) agar plates [30] to final concentrations of 0, 0.25, 0.5 and 1 mg/mL. Control plates were prepared with water and bacteria at the same proportions. Plates were incubated overnight at 37°C to allow bacteria growth. Synchronized L1 worms were cultured onto treatment plates in the presence or absence of aqueous extract and allowed to develop until the young adult stage at 20°C.

### 2.6. Bacterial growth curve

*E*. *coli* OP50 growth was evaluated over 4 h in the presence or absence of *Ilex paraguariensis* at 0.25, 0.5 or 1 mg/mL. The optical density was measured with a spectrophotometer at 600 nm. Growth curves were normalized with the control group at time zero [31].

### 2.7. ATGL-1::GFP expression

**Fig 1:**
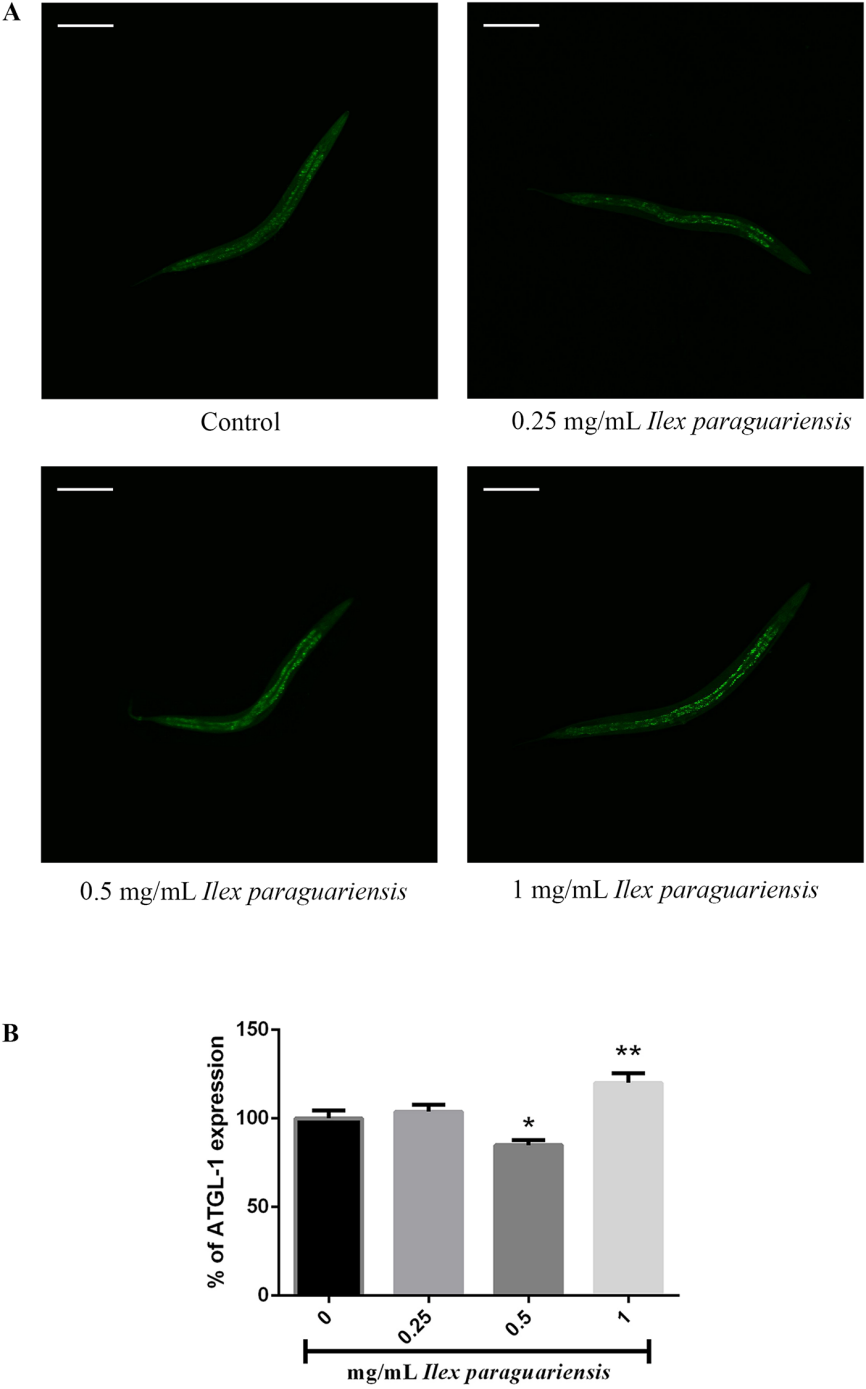
*Ilex paraguariensis* effects on ATGL-1::GFP expression in *Caenorhabditis elegans* VS20 strain. (A) Visualization of ATGL-1::GFP expression and (B) measurement of ATGL-1::GFP expression on young-adult *Caenorhabditis elegans*. *p<0.05 and **p<0.01, statistically significant compared with the untreated group by One-Way ANOVA followed by Bonferroni post-test (mean, standard error of the mean [SEM], *n* = 30 worms per group). The experiments were performed in triplicate.

To determine ATGL-1::GFP expression, young-adults worms from VS20 strain were immobilized with 10 mM sodium azide for image acquisition (Fig 1), and photographed under 60× objective on a confocal microscope (Fluoview FV101, Olympus, Tokyo, Japan). Images were processed with the Olympus Image Browser. GFP fluorescence quantification and analyses of images were conducted with ImageJ software by determining average pixel intensity per animal.

### 2.8. C1-BODIPY-C12 staining

**Fig 2:**
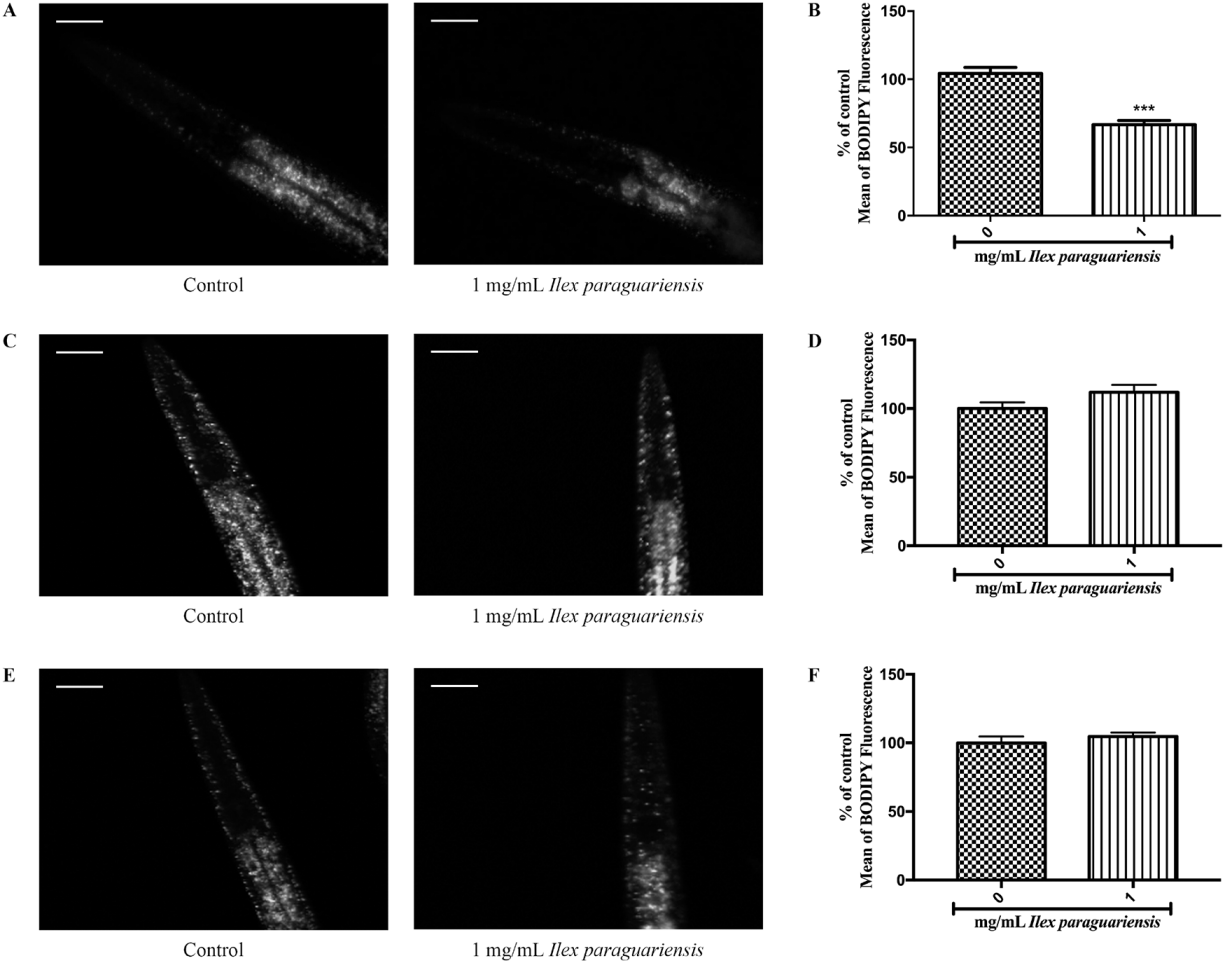
BODIPY fluorescence decrease induced by *Ilex paraguariensis* exposition in *Caenorhabditis elegans* wild-type (N2). Visualization of lipid droplets evidenced by BODIPY labeling in (A) wild-type young adults, (B) *nhr-49(nr2041)*, and (C) *ador-1(ox489)* and fluorescence quantification in (D) wild-type young adults, (E) *nhr-49(nr2041)*, and (F) *ador-1(ox489)*. ***p<0.001, statistically significant compared with the untreated group by Student’s *t*-test (mean, SEM, *n* = 60 worms per group). The experiment was performed in triplicate.

C1-BODIPY-C12 conjugated fatty acids (BODIPY) lipid staining was carried out as previously described [32]. C1-BODIPY-C12 was applied to the surface of NGM plates (10 mL agar) seeded with *E*. *coli* OP50 and 0 or 1 mg/mL of *Ilex paraguariensis* to a 50 nM final concentration (Fig 2 or S1 Fig). Synchronized wild-type (N2), *nhr-49(nr2041)*, and *ador-1(ox489)* L1-stage worms were transferred to these plates and allowed to develop until adulthood. Young-adult worms were mounted on agar pads and immobilized with 10 mM sodium azide for image acquisition using identical settings and appropriate filters with a Zeiss Axiovert II microscope (Thornwood, NY, USA) fitted with a CCD camera. Fluorescence quantification and analyses of images were conducted with ImageJ software by determining average pixel intensity of the two first intestinal pairs of cells per animal.

### 2.9. Pharynx pumping rate

**Fig 3:**
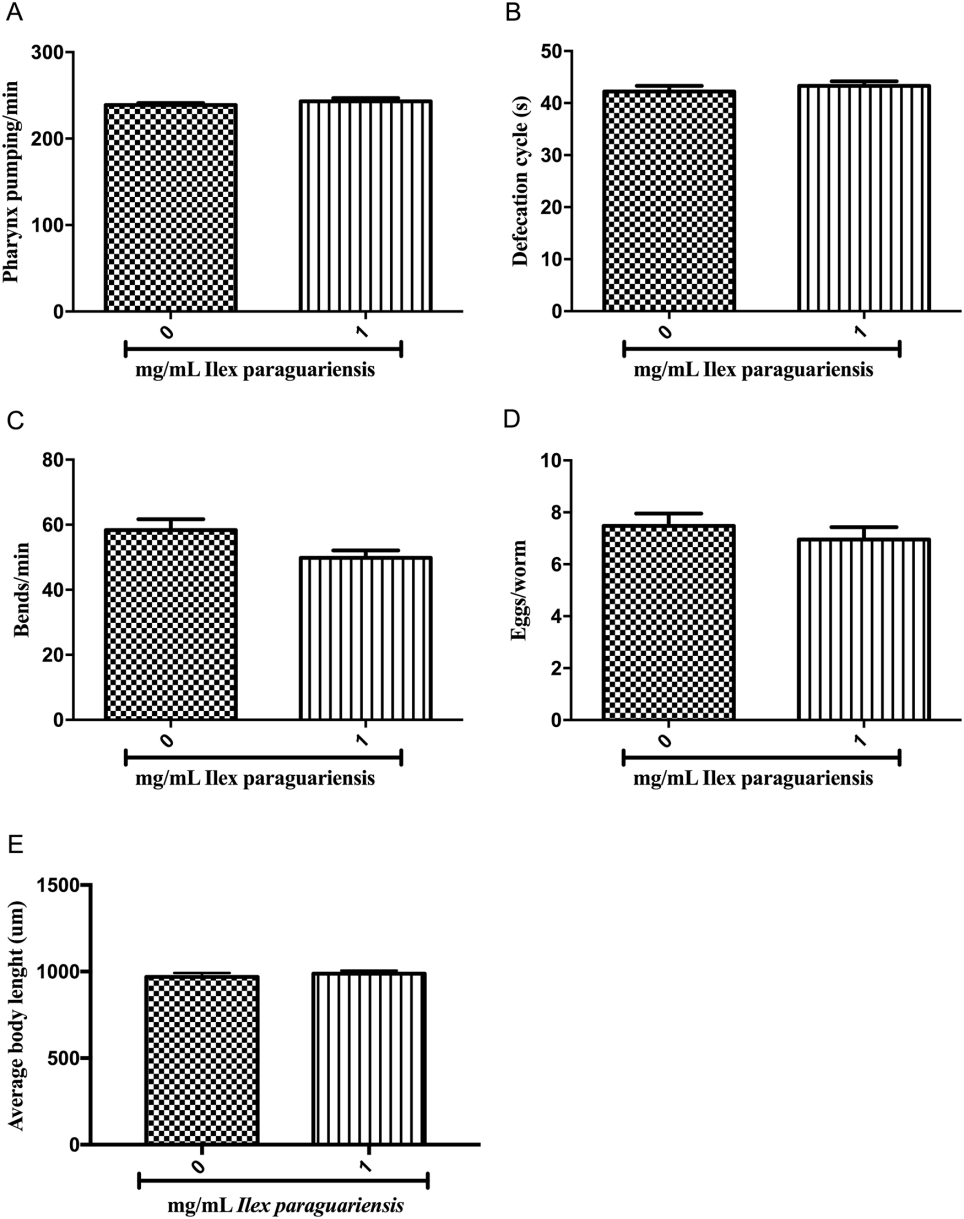
*Caenorhabditis elegans* wild-type behavior following *Ilex paraguariensis* treatment. Effect of *I*. *paraguariensis* on (A) pharyngeal pumping rate, (B) defecation cycle length, (C) body bends in wild-type young adults, (D) egg production and (E) body length in wild-type young-adults. No statistically significant difference was found by Student’s *t*-test (mean, SEM, *n* = 30 worms per group). The experiments were performed in triplicate.

Pharyngeal bulb contractions were measured in young adult worms on their treatment plates. The number of pharynx pumps in a 10s-interval, in triplicate [33], was assessed with a microscope (Fig 3A).

### 2.10. Defecation assay

Defecation frequencies were performed by observing young adult worms in their plates of treatment [34] with a microscope. The defecation cycle length was defined as the duration between the pubic steps (posterior body muscle contraction) of two consecutive defecations (Fig 3B).

### 2.11. Body bends frequency

After treatment, young adult worms were randomly transferred to food-free NGM plates and allowed to freely move for 3 min to adaptation. The number of times each worm changes the direction of the body was scored with a microscope during a 20s-interval in triplicate [24] (Fig 3C).

### 2.12. Egg-production

To assess the number of eggs inside the uterus, young-adult worms were individually picked into a drop of bleaching solution (1% NaOCl, 0.25 M NaOH). The worms’ cuticles were disrupted and released eggs were counted with a microscope [35] (Fig 3D).

### 2.13. Developmental evaluation

To evaluate the development of wild-type animals following *Ilex paraguariensis* treatment, images of worms from each group were acquired (Fluoview FV101, Olympus, Tokyo, Japan). Images were processed with the Olympus Image Browser and body length was measured along the animal axis using ImageJ software (Fig 3E).

### 2.14. Oxygen consumption

**Fig 4:**
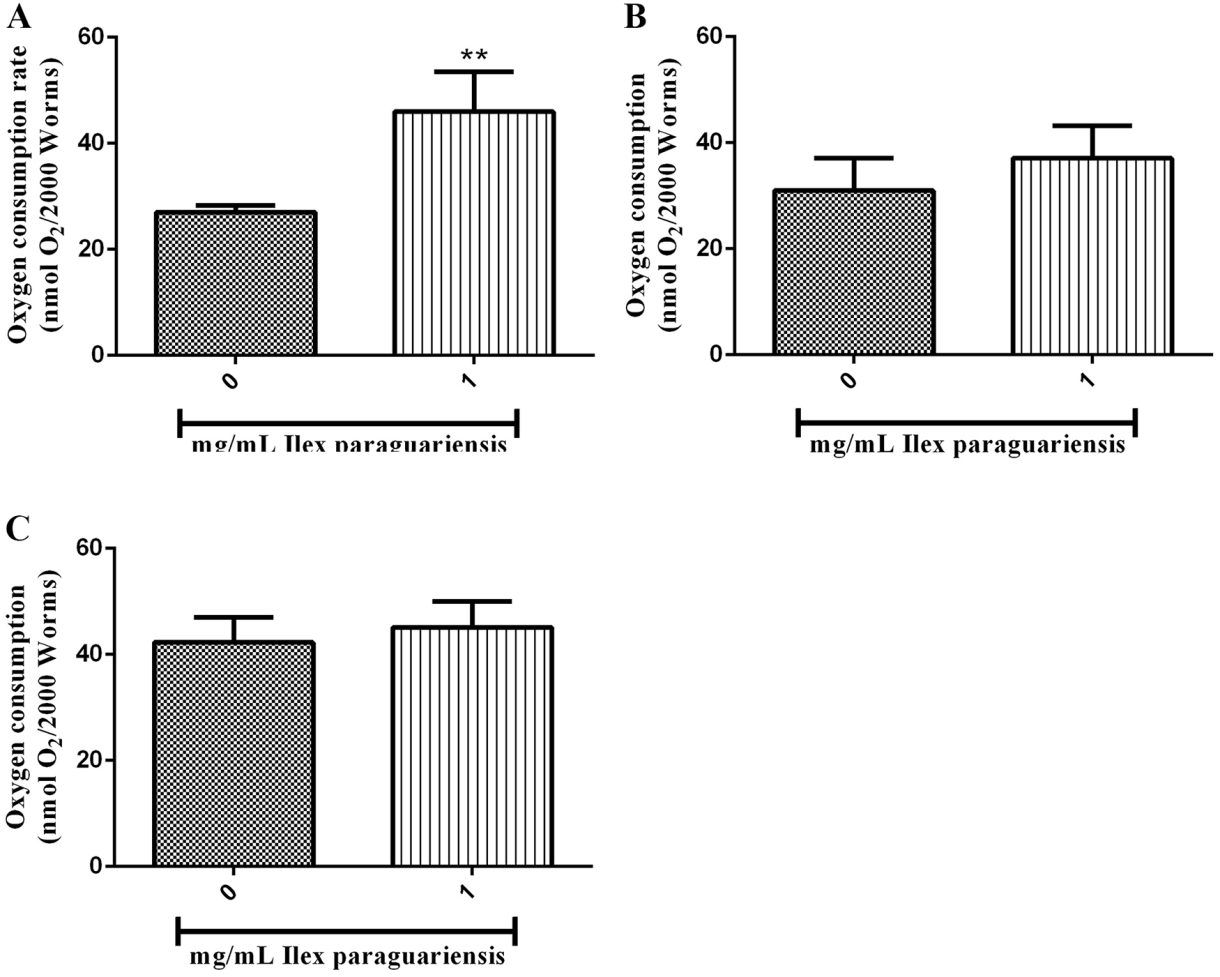
Measurement of oxygen consumption rate in *C*. *elegans* treated with *I*. *paraguariensis* extract. Oxygen consumption rates in young-adult worms. (A) Wild type, (B) *nhr-49(nr2041)*, and (C) ador-1(ox489). **p < 0.01, statistically significant compared with the untreated group by Student’s *t*-test (mean, SEM, *n* = 7). The experiments were performed seven times.

Oxygen consumption rate was measured with a Hansatech Oxymeter (Pentney, Norfolk, UK) with a Clark-type electrode. The electrode chamber was washed and stabilized for 30 min with 1 mL air-saturated M9 buffer before analysis. Approximately 2,000 young-adult worms of wild-type (N2), *nhr-49(nr2041)*, and *ador-1(ox489)* were transferred to a cuvette with 1 mL of M9, and oxygen consumption was measured for 2–15 min at 20°C to obtain oxygen consumption rates [36] (Fig 4).

### 2.15. Oxidative stress resistance assays

**Fig 5:**
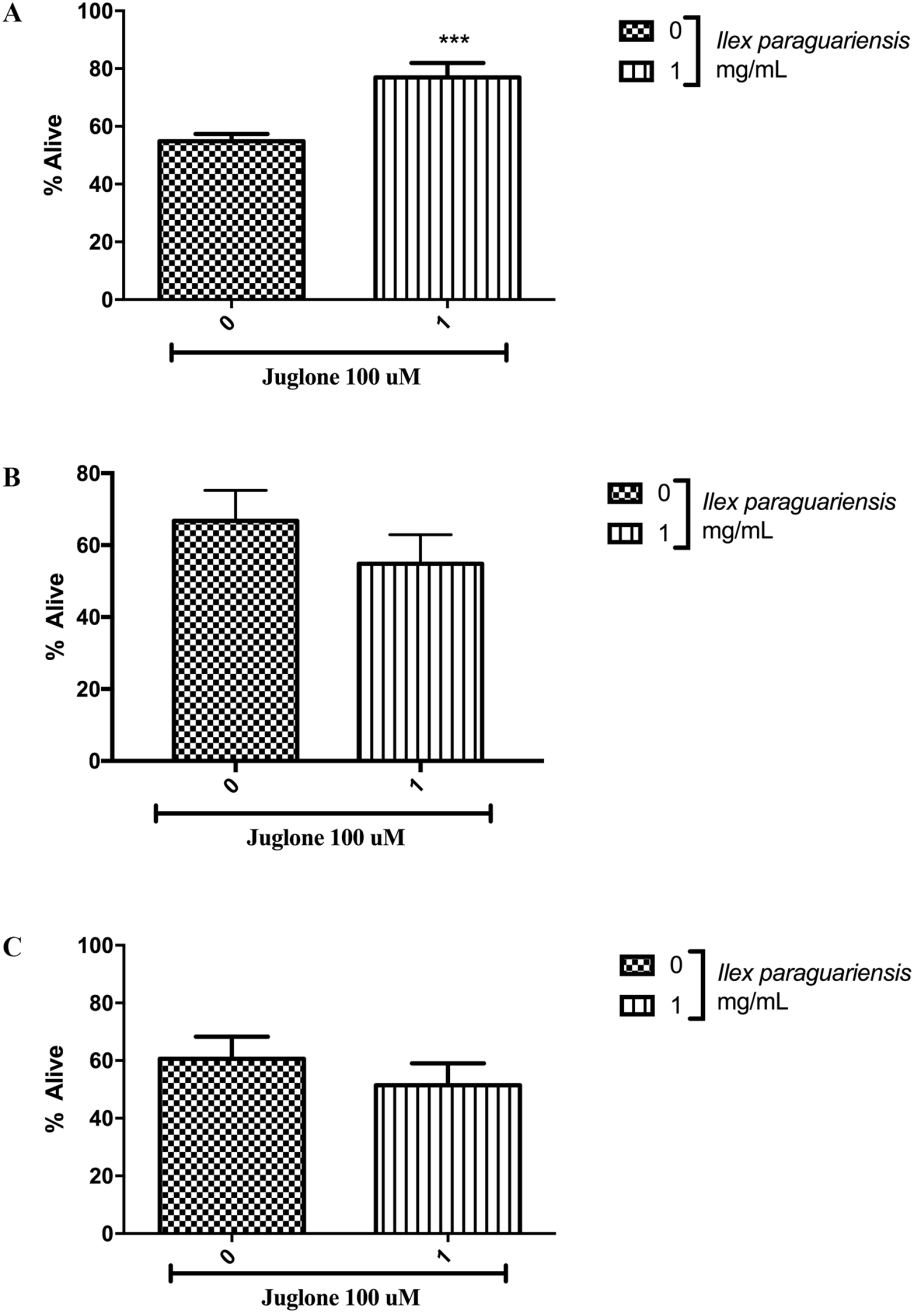
*I*. *paraguariensis* on resistance to oxidative stress. Survival of young-adult worms exposed to 100 μM juglone for 1 h. (A) Wild type, (B) *nhr-49(nr2041)*, and (C) *ador-1(ox489)*. Data are expressed as percentage of worms alive. **p < 0.01, statistically significant compared with the untreated group by Student’s *t*-test (mean, SEM, *n* = approximately 500 worms per group).

Young-adult worms of wild-type (N2), *nhr-49(nr2041)*, and *ador-1(ox489)* were exposed to 100 uM of juglone, also called 5-hydroxy-1,4- naphthoquinone (IUPAC), a generator of reactive oxygen species (ROS) [37], this concentration is supposed to kill approximately 50% of the nematodes (LD50), after 1 hour exposure [38]. Juglone was prepared in EtOH (1% final concentration). After 1 hour at 20°C, 100 nematodes per treatment with *Ilex paraguariensis* were assessed with a Nikon E200 microscope (Tokyo, Japan). Animals that reacted to a mechanical stimulus were scored as alive, and non-responding animals were considered to be dead (Fig 5). Analyses were carried out in five independent assays. Results are shown as percentage of alive animals.

### 2.16. Fluorescence measurement of *hsp-16*.*2p*::GFP

**Fig 6:**
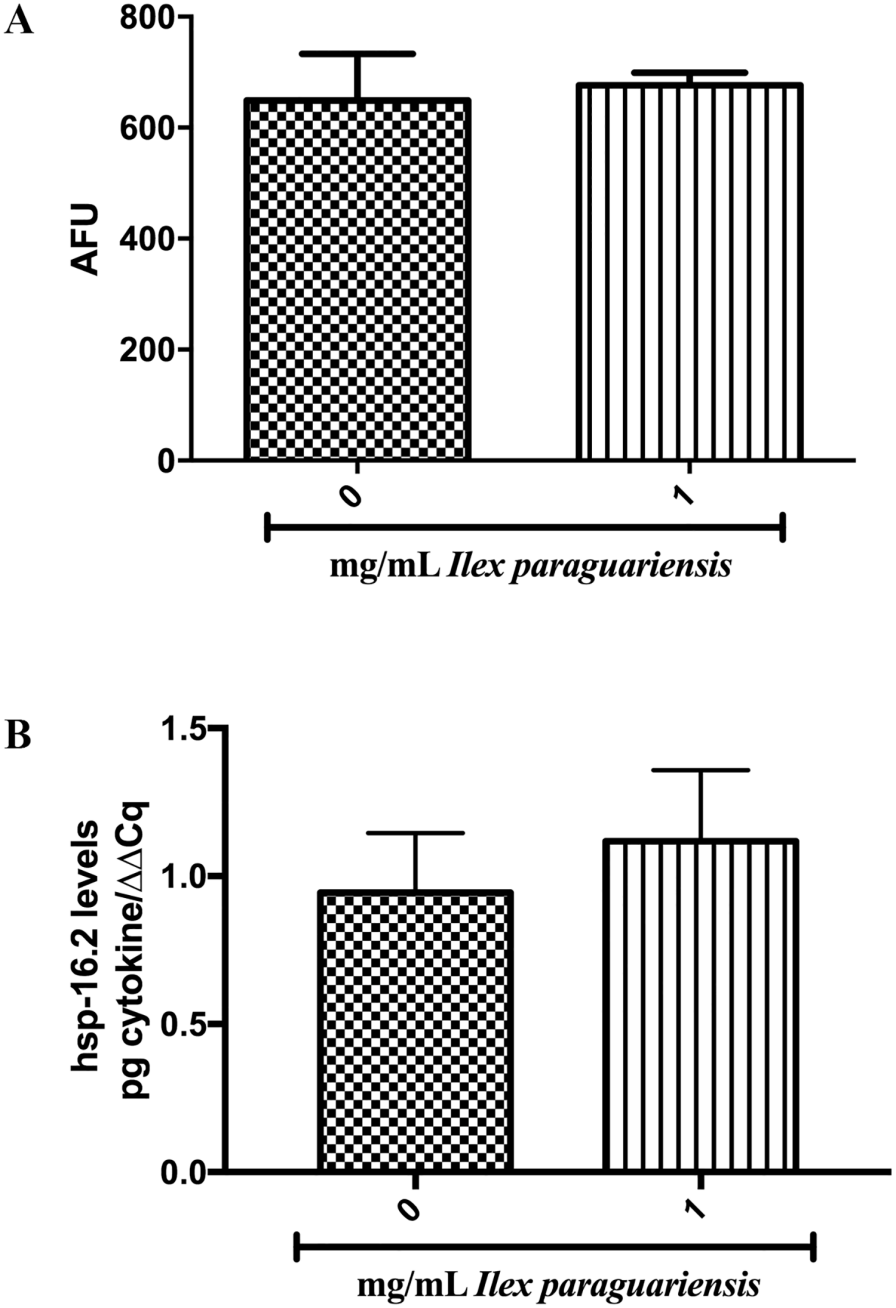
*I*. *paraguariensis* on *hsp-16*.*2p*::GFP and *hsp-16*.*2* transgene expression. (A) Fluorescence expression of *hsp-16*.*2p*::GFP worms. Data are expressed as arbitrary fluorescence units (AFU). (B) *hsp-16*.*2* mRNA levels using a ΔΔCq method in wild-type (N2). Student’s *t*-test, p < 0.05. The experiments were performed five times on different days.

Fluorescence measurement of *hsp-16*.*2p*::GFP was measured at emission 535 nm and excitation 485 nm with 200 worms at young-adult stage [39], using SpectraMax^®^ i3x microplate reader (Molecular Devices, Sunnyvale, CA). Analyses were carried out in triplicate and repeated independently five times (Fig 6A).

### 2.17. Quantitative real-time polymerase chain reaction (qPCR)

The relative abundance of small heat shock protein (sHSP) *hsp-16*.*2* mRNA was measured in the N2 wild-type strain by quantitative real-time PCR (qPCR) following a described method with some modifications [40]. The animals were washed with M9 buffer into Eppendorf tubes, resuspended in Trizol reagent (Invitrogen) and allowed to settle on ice. The worm pellet was followed by chloroform extraction and isopropanol precipitation. Total RNA isolation was performed accordingly to the manufacturer’s suggested protocol.

Total RNA samples were treated with DNase I (Promega) to eliminate DNA contamination. Reverse transcription (RT) of approximately 1 μg total RNA was performed with random primer, dNTPs and M-MLV reverse transcriptase enzyme (Invitrogen), according to the manufacturer’s suggested protocol. The following gene-specific primers were used: Actin (Forward—5'-GTGTGACGACGAGGTTGCCGCTCTTGTTGTAGAC-3' and Reverse—5'-GGTAAGGATCTTCATGAGGTAATCAGTAAGATCAC-3'), and *hsp-16*.*2* (Forward—5'-CTGCAGAATCTCTCCATCTGAGTC-3' and Reverse—5'-AGATTCGAAGCAACTGCACC-3') in 10 μl PCR mixture containing 5-μl cDNAs (1:100), 1 X PCR Buffer, 0,1 mM dNTPs, 0.2 μM of each primer, 3 mM MgCl_2_, 0.1 X SYBR Green I (Molecular Probes) and 0,5 U Platinum *Taq* DNA Polymerase (Invitrogen).

The qPCR conditions were: 94 °C for 5 min followed by 40 cycles of 15 s at 94 °C, 15 s at 60 °C and 40 s at 72 °C for extension in a Thermocycler StepOne Plus (Applied Biosystems). After amplification, samples were heated from 60 to 95 °C at a 0.3 °C/s temperature gradient to construct the denaturing curve of the amplified products. All samples were analyzed in triplicate with a non-template control also included. SYBR Green fluorescence (Molecular probes) was analyzed by StepOne Plus Software version 2.0 (Applied Biosystems) and Cq value (ΔCq) for each sample was calculated and reported using the ΔΔCq method [41]. Briefly, for each well, a ΔCq value was obtained by the difference in Cq values (ΔCq) between the target gene and the reference gene. The ΔCq mean value obtained from the control group of each gene was used to calculate the ΔΔCq of the respective gene (2^-ΔΔCq^) (Fig 6B).

### 2.18. Statistical analyses

Statistical analyses were performed using GraphPad Prism 5.0 (GraphPad Software, San Diego, CA). The statistical differences between conditions were determined by a by Student’s T-test, while the statistical differences between different concentrations of *Ilex paraguariensis* were determined by a one-way ANOVA followed by Bonferroni’s *post-hoc* test. Values are represented as means ± SEM, results were considered statistically significant when p < 0.05.

## 3. Results

### 3.1. High performance liquid chromatography analysis of *I*. *paraguariensis* the bioactive compounds

High performance liquid chromatography fingerprinting of *Ilex paraguariensis* aqueous extracts revealed the presence of gallic acid (t_R_ = 9.97 min; peak 1), catechin (t_R_ = 15.03 min; peak 2), chlorogenic acid (t_R_ = 21.38 min; peak 3), caffeic acid (t_R_ = 24.19 min; peak 4), caffeine (t_R_ = 27.48 min; peak 5), theobromine (t_R_ = 32.65 min; peak 6), epigallocatechin (t_R_ = 35.11 min; peak 7), rutin (t_R_ = 39.45 min; peak 8), quercetin (t_R_ = 49.27 min; peak 9), and kaempferol (t_R_ = 54.67 min; peak 10) (Table 1).

### 3.2. Antimicrobial effect of *Ilex paraguariensis*

Since dead bacteria can provide differences in micronutrients and be responsible for some differences in the range of fat stores in C. elegans [42, 43], the antimicrobial activity of I. paraguariensis extract against Escherichia coli OP50 was tested. No differences were observed in bacterial growth in the presence of 0.25, 0.5, or 1 mg/mL of I. paraguariensis (data not shown).

### 3.3. Effects of *Ilex paraguariensis* on ATGL-1 expression and *C*. *elegans* fat storage

We determined a concentration of *I*. *paraguariensis* extract that increased the expression of ATGL-1 fused with green fluorescent protein (ATGL-1::GFP). We observed that 1 mg/mL of *I*. *paraguariensis* extract increased the expression of ATGL-1::GFP (20.06%) in the VS20 strain (Fig 1, p < 0.05). No differences were observed between 0.25 mg/mL and control groups, but 0,5 mg/mL decreased the expression of ATGL-1::GFP (15.19%). Moreover, BODIPY, a proxy for the measurement of fat in *C*. *elegans*, was used to evaluate structures in gut epithelial and hypodermal cells. *Ilex paraguariensis* treatment decreased (37.57%) the fluorescence levels of N2 wild-type worms compared with the control group (Fig 2A and 2D, p < 0.001), however, when the *nhr-49(nr2041)* and *ador-1(ox489)* strains were treated, no differences were observed in the fluorescence levels compared with control group (Fig 2B, 2C, 2E and 2F).

### 3.4. Developmental and behavior of *I*. *paraguariensis*-treated worms

Treatment with *I*. *paraguariensis* extract did not affect the pharynx-pumping rate and defecation cycle length of adult wild-type worms. Both parameters were similar to control groups (Fig 3A and 3B). The rate of movement was assessed through the frequency of body bends, and no difference was observed (Fig 3C). Egg production was also assessed in young-adult worms following extract treatment and there was no difference between control and treated worms (Fig 3D). Moreover, there was no difference in body length between control and treated worms (Fig 3E).

### 3.5. Oxygen consumption in *C*. *elegans*

The oxygen consumption rate was compared between untreated and treated worms with 1 mg/mL of *I*. *paraguariensis* of wild-type (N2), *nhr-49(nr2041)*, and *ador-1(ox489)* strains to infer body energy expenditure. In the N2-treated group, the oxygen consumption rate was increased by 70.16% compared with control animals (Fig 4A, p < 0.05). By contrast, when the *nhr-49(nr2041)* and *ador-1(ox489)* strains were treated, no differences were observed in oxygen consumption rate compared with the control group (Fig 4B and 4C).

### 3.6. Effect of *I*. *paraguariensis* on resistance to oxidative stress

We analyzed if *I*. *paraguariensis* extract could reduce the oxidative damage caused by juglone, which generates reactive oxygen species (ROS) [37]. The 1-h exposure of young adult N2 wild-type worms to 100 mM of juglone killed 45.16% of worms, and pretreatment with 1 mg/mL of *I*. *paraguariensis* extract increased worm survival following juglone exposure by 22.12% (Fig 5A, p < 0.001), however, when the *nhr-49(nr2041)* and *ador-1(ox489)* strains were treated, no differences were observed in worm survival compared with control group (Fig 5B and 5C). Following this, we determined the expression of *hsp-16*.*2p* in CL2070 worms and the relative abundance of *hsp-16*.*2* mRNA in N2 wild-type worms. The treatment with 1mg/mL of *I*. *paraguariensis* extract did not alter *hsp-16*.*2p* expression in CL2070 worms, neither the *hsp-16*.*2* mRNA levels in N2 worms (Fig 6).

## 4. Discussion

The main findings of this study indicate that treatment with 1 mg/mL of *I*. *paraguariensis* extract increased worm survival following oxidative stress, reduced fat storage, increased *atgl-1(nr2041)* expression and oxygen consumption in N2 wild-type. Taken together, our results clearly indicate that *I*. *paraguariensis* extract can protect against oxidative stress and modulate fat metabolism by increasing *atgl-1(nr2041)* expression and oxygen consumption rate in *C*. *elegans*. Possible pathways include expression of the *nhr-49* gene, a gene linked to β-oxidation and *ador-1*, a gene that encodes an ortholog of the human adenosine receptor. The effects on fat metabolism appear to be unrelated with any behavioral procedure tested here, but are important for survival, furthermore, the expression of HSPs seems to be unrelated to the effects on metabolism or survival.

The concentrations of *I*. *paraguariensis* used here did not present antimicrobial activity, thus did not affect the *E*. *coli* growth [42, 43]. Moreover, the animals were observed through the larval stages using a microscope, and all animals achieve the young adult stage at the same time, and the same length, so it does not alter the development time.

The metabolism of fatty storage is regulated by the synthesis and degradation of fat, mainly TAG, the storage form of carbohydrate and fat, depicting a highly concentrated form of energy [44]. The TAG storage and mobilization are general biological process of cells, defined as lipogenesis and lipolysis, respectively. The lipogenesis process includes fatty acid synthesis and subsequent TAG synthesis, and occurs when the total available energy is not immediately needed for metabolic processes, so, the energy contained in Acetyl-CoA is efficiently stored for a long time as fat [45]. In contrast, lipolysis encompasses the catabolism of TAG stored into lipid droplets, to mobilize stored energy [45].

Since the intestine is a major fat storage organ in *C*. *elegans* [46], and TAGs are the major lipids stored in the organism, we determined a concentration of *I*. *paraguariensis* extract that would modulate key factors of fat metabolism without suppressing bacterial growth. To mobilize fat stored, TAGs must be hydrolyzed, and this reaction requires two major enzymes, the adipose triglyceride lipase (ATGL), and the hormone sensitive lipase (HSL) [47]. Here, we evaluated the expression of *atgl-1(nr2041)*, the major lipase for fat mobilization from TAGs stored in *C*. *elegans* [26]. We found that 1 mg/mL of *I*. *paraguariensis* extract increased *atrgl-1(nr2041)* expression (Fig 1). This result indicates that the extract, at 1 mg/mL, can act in fat metabolism of *C*. *elegans*, increasing fat mobilization from storage.

Remarkably, 0.5mg/mL of *I*. *paraguariensis* extract decreased *atgl-1(nr2041)* expression (Fig 1) of *C*. *elegans*. This effect appears to be caused by a biphasic effect of *I*. *paraguariensis* extract. This kind of effect is well established in pharmacology studies as the U-shaped plot [48]. So considering the *I*. *paraguariensis* extract action at the *atgl-1(nr2041)* expression–lower concentration has no effect in *atgl-1(nr2041)* expression, while our medium concentration reduced the expression and our higher concentration tested here increased the *atgl-1(nr2041)* expression–it could be interpreted as an "U-shaped plot”.

Since *C*. *elegans* can convert excess energy into TAGs stored in lipid droplets and distributed throughout the body of the worm [49], we assessed fat storage through BODIPY-conjugated fatty acid staining. Despite being a simple procedure, it is reliable, inexpensive, does not require fixation [50, 51], and allowed us to investigate both intestinal and hypodermal lipid stores of live animals. We found that 1 mg/mL of *I*. *paraguariensis* decreased BODIPY fluorescence in N2 wild-type (Fig 2A and 2D). Therefore, 1 mg/mL of *I*. *paraguariensis* extract was the primary concentration tested, since it did not affect bacterial growth, increased ATGL-1::GFP expression, and decreased BODIPY fluorescence in N2 wild-type (Figs 1 and 2). The increased expression of *atgl-1(nr2041)* is consistent with the decrease in fat storage.

Afterwards, we speculate if the decrease in fat storage could be caused by a reduction in food intake [52], or an increase in the defecation cycle. Our results (Fig 3A and 3B) indicate that the decrease in fat storage was not related to pharyngeal pumping or the defecation cycle rate. Alternatively, body movements are also associated with changes in fat storage, the increase in movement may increase the usage of energetic reserves, leading to a decrease in fat storage [53]. In addition, increased egg production can also indirectly alter fat metabolism, because in oocyte production, polyunsaturated fatty acids are transported from the site of fat metabolism [54]. The results (Fig 3C and 3D) show that the decrease in fat storage was not related to body movements or egg production. Thus, the increase in fat mobilization or decrease in fat storage induced by *I*. *paraguariensis* seems to be unrelated to any of the changes in the behavioral parameters tested in this study.

We demonstrate here that *I*. *paraguariensis* extract increased the oxygen consumption rate of the N2 strain (Fig 4A), and increased β-oxidation (*nhr-49* modulation) promoting an acetyl-CoA availability for the mitochondrial tricarboxylic acid (TCA) cycle and other compounds that feed the electron transport chain [55]. In order to attend this demand for energy, *C*. *elegans* increases the mobilization of fatty acids (*atgl-1*) and decrease the fat storage. All of this effects could be associated with an increase in uncoupling proteins (UCPs) [56, 57], mitochondrial transporters present in the inner membrane of mitochondria responsible for thermogenesis [58], that occurs independently of behavioral alterations. This modulation caused by the extract in the oxygen consumption brings the modulations of fat oxidation and consequently a decrease in fat storage.

To test the involvement of fatty acid oxidation in the lipid-reducing effects of *I*. *paraguariensis* extract, we investigated the nuclear hormone receptor, *nhr-49*, a key regulator gene of fat oxidation in *C*. *elegans* [59]. *nhr-49* targets multiple enzymes involved in β-oxidation [25], and it was previously demonstrated that *nhr-49(nr2041)* animals have higher fat content [25]. The *I*. *paraguariensis* extract did not alter BODIPY fluorescence and neither the oxygen consumption rate in *nhr-49(nr2041)* worms (Figs 2B and 4B). Therefore, the oxygen consumption increase and BODIPY fluorescence decrease in wild-type worms is related to the NHR-49 pathway.

Previous studies have reported that the main compounds identified in the aqueous extract of *I*. *paraguariensis* are methylxanthines, mainly caffeine [13, 14]. Caffeine is a well-known thermogenic agent related to increased metabolic rates (for a review of the effects of caffeine, see Harpaz, Tamir [60]). The primary action of caffeine and other methylxanthines occurs via antagonism of the adenosine receptor, resulting in phosphodiesterase inhibition and increased lipolysis [61]. Interaction of the adenosine system and caffeine has already been described [62], and the first knockout of purinergic receptors was recently generated [63]. To confirm that the purinergic system is involved in the effects induced by the extract, oxygen consumption and BODIPY fluorescence of the *ador-1* knockout strain were assessed. Similar to the experiment with *nhr-49(nr2041)*, *I*. *paraguariensis* did not alter BODIPY fluorescence and neither the oxygen consumption rate of the *ador-1(ox489)* (Figs 2C and 4C), indicating that the purinergic system is involved in the effects observed in N2 worms.

Increased oxygen consumption leads to an increase in ROS formation [64]. In general, plant extracts containing phenolic compounds exhibit intrinsic antioxidant activity or induce antioxidant pathways [65]. A previous study has already shown that *I*. *paraguariensis* extract reduced ROS levels and increased *C*. *elegans* survival [66]. Here, we exposed N2, *ador-1(ox489)* and *nhr-49(nr2041)* worms treated with 1 mg/mL *I*. *paraguariensis* to a chemical generator of ROS, juglone [37], to evaluate the potential antioxidant effects of *Ilex paraguariensis*. The extract protected wild-type *C*. *elegans*, but not *ador-1(ox489)* neither *nhr-49(nr2041)* against the pro-oxidant effects of juglone (Fig 5).

Goh and cols have already demonstrated that NHR-49 is not only a regulator of lipid metabolism, but also it is required for the activation of a protective transcriptional response to oxidative stress [67]. Here we showed that *nhr-49(nr2041)* is required for juglone resistance. Since fat accumulation is correlated with systemic oxidative stress in humans and mice [68], we demonstrated that 1 mg/mL of *I*. *paraguariensis* extract can protect *C*. *elegans* wild-type against the production of ROS by juglone through NHR-49 pathway and purinergic system.

To test if HSPs, a group of low molecular weight polypeptides induced by environmental and physiological stress [69], are involved in the oxidative stress protection induced by *I*. *paraguariensis* extract, we assessed *hsp-16*.*2p* expression in CL2070 worms and relative abundance of *hsp-16*.*2* mRNA in N2 wild-type worms. We observed that *hsp-16*.*2* is not involved in the *I*. *paraguariensis* stress response (Fig 6). Here, we showed that *I*. *paraguariensis* extract has thermogenic properties and promotes fat oxidation and oxidative stress protection independent of *hsp-16*.*2* activation.

The *I*. *paraguariensis* extract has been linked to various biological activities, which have been mainly attributed to its large amount of bioactive compounds, including the methylxanthines caffeine and theobromine and the phenolic compounds caffeic acid, chlorogenic acid, and saponins [70]. The use of isolated compounds or active principle(s) from natural extracts is encouraged by some researchers [71, 72]; nevertheless, this may not be the most effective method in all cases. For example, Dulloo and colleagues have already shown in both *in vitro* and *in vivo* studies that the increase in thermogenic effects induced by green tea was lost when the same quantity of isolated caffeine present in the extract was tested [73, 74].

Synergistic interactions are important in phytomedicines, therefore, the effects of the *I*. *paraguariensis* extract on adipose tissue could be lost when a single active ingredient is isolated and used at low concentrations [75]. In some cases, isolated compounds of plant extracts can exert the same effect only at higher concentrations, which might be toxic to the organism [76]. Thus, the results mentioned above of *Ilex paraguariensis* extract could be lost when a single active ingredient is isolated at the same concentration found in the extract.

To the best of our knowledge, this is the first demonstration of a decrease in *C*. *elegans* fat storage following treatment with *I*. *paraguariensis* extract. Since *nhr-49* targets multiple enzymes involved in β-oxidation [25], this effect could be caused by an increase in the β-oxidation pathway through the *nhr-49* gene, inducing overexpression of *atgl-1 gene*. *atgl-1* could increase the oxygen consumption rate in the respiratory chain, possibly due to an increase in TCA cycle products, independently of behavioral alterations related to energy expenditure. Additionally, these effects could also be associated with an increase in *hosl-1* (hormone-sensitive lipase orthologue) expression, since *hosl-1* overexpression is related with the decreased of fat accumulation in *C*. *elegans* [77, 78].

Furthermore, we clearly demonstrate that the purinergic system is involved in the increased oxygen consumption rate of *C*. *elegans*, and this effect was *ador-1*-dependent. In addition to metabolic effects, the extract also exhibited an antioxidant effect that protected *C*. *elegans* against the production of ROS, increasing the survival of worms exposed to juglone in an HSP-independent manner. These results suggest that *C*. *elegans* is a reasonable model for screening the effects of natural products on lipid metabolism.

## Supporting information

S1 Fig Representative images of lipid accumulation through C1-BODIPY-C12 lipid staining in (A) N2, (B) *nhr-49*, and (C) *ador-1* worms. (TIF)
