# Supplementary figures and images for: Ilex paraguariensis modulates fat metabolism in Caenorhabditis elegans through purinergic system (ADOR-1) and nuclear hormone receptor (NHR-49) pathways
Source: PLoS One. 2018 Sep 25;13(9):e0204023. doi: 10.1371/journal.pone.0204023 (PMC6155532; doi:10.1371/journal.pone.0204023)

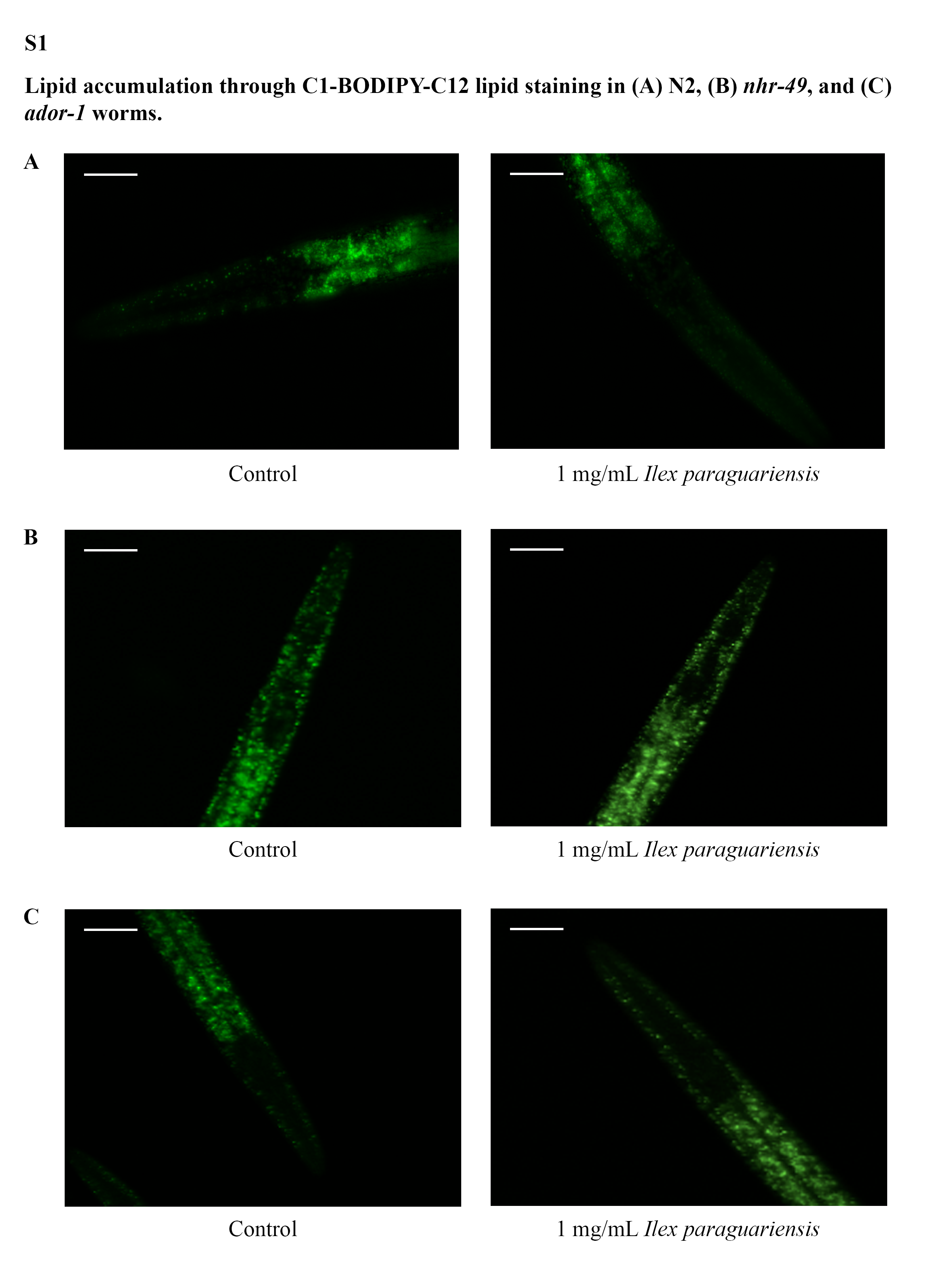

Supplement: S1 Fig — (TIF) [file pone.0204023.s001.tif]
